# Acute Simvastatin Inhibits K**_ATP_** Channels of Porcine Coronary Artery Myocytes

**DOI:** 10.1371/journal.pone.0066404

**Published:** 2013-06-17

**Authors:** Sai Wang Seto, Alice Lai Shan Au, Christina Chui Wa Poon, Qian Zhang, Rachel Wai Sum Li, John Hok Keung Yeung, Siu Kai Kong, Sai Ming Ngai, Song Wan, Ho Pui Ho, Simon Ming Yuen Lee, Maggie Pui Man Hoi, Shun Wan Chan, George Pak Heng Leung, Yiu Wa Kwan

**Affiliations:** 1 The Vascular Biology Unit, Queensland Research Centre for Peripheral Vascular Disease, School of Medicine and Dentistry, James Cook University, Townsville, Queensland, Australia; 2 School of Biomedical Sciences, Faculty of Medicine, The Chinese University of Hong Kong, Shatin, Hong Kong, PR of China; 3 Department of Pharmacology and Pharmacy, Faculty of Medicine, The University of Hong Kong, Hong Kong, PR of China; 4 State Key Laboratory of Chinese Medicine and Molecular Pharmacology, Department of Applied Biology and Chemical Technology, The Hong Kong Polytechnic University, Kowloon, Hong Kong, PR of China; 5 School of Life Sciences, Faculty of Science, The Chinese University of Hong Kong, Shatin, Hong Kong, PR of China; 6 Department of Surgery, Faculty of Medicine, The Chinese University of Hong Kong, Shatin, Hong Kong, PR of China; 7 Department of Electronic Engineering, Faculty of Engineering, The Chinese University of Hong Kong, Shatin, Hong Kong, PR of China; 8 Institute of Chinese Medical Sciences, the University of Macau, Macau, PR of China; Texas A & M, Division of Cardiology, United States of America

## Abstract

**Background:**

Statins (3-hydroxy-3-methyl-glutaryl coenzyme A (HMG-CoA) reductase inhibitors) consumption provides beneficial effects on cardiovascular systems. However, effects of statins on vascular K_ATP_ channel gatings are unknown.

**Methods:**

Pig left anterior descending coronary artery and human left internal mammary artery were isolated and endothelium-denuded for tension measurements and Western immunoblots. Enzymatically-dissociated/cultured arterial myocytes were used for patch-clamp electrophysiological studies and for [Ca^2+^]_i_, [ATP]_i_ and [glucose]_o_ uptake measurements.

**Results:**

The cromakalim (10 nM to 10 µM)- and pinacidil (10 nM to 10 µM)-induced concentration-dependent relaxation of porcine coronary artery was inhibited by simvastatin (3 and 10 µM). Simvastatin (1, 3 and 10 µM) suppressed (in okadaic acid (10 nM)-sensitive manner) cromakalim (10 µM)- and pinacidil (10 µM)-mediated opening of whole-cell K_ATP_ channels of arterial myocytes. Simvastatin (10 µM) and AICAR (1 mM) elicited a time-dependent, compound C (1 µM)-sensitive [^3^H]-2-deoxy-glucose uptake and an increase in [ATP]_i_ levels. A time (2–30 min)- and concentration (0.1–10 µM)-dependent increase by simvastatin of p-AMPKα-Thr^172^ and p-PP2A-Tyr^307^ expression was observed. The enhanced p-AMPKα-Thr^172^ expression was inhibited by compound C, ryanodine (100 µM) and KN93 (10 µM). Simvastatin-induced p-PP2A-Tyr^307^ expression was suppressed by okadaic acid, compound C, ryanodine, KN93, phloridzin (1 mM), ouabain (10 µM), and in [glucose]_o_-free or [Na^+^]_o_-free conditions.

**Conclusions:**

Simvastatin causes ryanodine-sensitive Ca^2+^ release which is important for AMPKα-Thr^172^ phosphorylation via Ca^2+^/CaMK II. AMPKα-Thr^172^ phosphorylation causes [glucose]_o_ uptake (and an [ATP]_i_ increase), closure of K_ATP_ channels, and phosphorylation of AMPKα-Thr^172^ and PP2A-Tyr^307^ resulted. Phosphorylation of PP2A-Tyr^307^ occurs at a site downstream of AMPKα-Thr^172^ phosphorylation.

## Introduction

3-Hydroxy-3-methyl-glutaryl coenzyme A (HMG-CoA) reductase is a 97-kDa glycoprotein embedded in the endoplasmic reticulum [1] which is involved in the endogenous cholesterol biosynthesis in mammalian liver and intestine [2]. Pervious study of our group [3] has clearly illustrated the biochemical existence of extra-hepatic HMG-CoA reductase in human and porcine cardiovascular tissues, suggesting a physiological role of this enzyme in the cardiovascular system. HMG-CoA reductase inhibitors, commonly known as statins, have been shown to be an effective treatment of hypercholesterolemia and cardiovascular diseases via its cholesterol-lowering property and cholesterol-independent effects (pleiotropic effects) [3], [4], [5], [6], [7], [8].

Regulation of vascular tone relies on complex cellular mechanisms as well as the opening and closing of various ion channels. Previous studies have demonstrated that statins can modify the activities of different ion channels in blood vessels including L-type Ca^2+^ channel and BK_Ca_ channel [3], [9], [10], [11]. In addition to Ca^2+^ channels and BK_Ca_ channels, ATP-sensitive K^+^ (K_ATP_) channels are abundant in vascular tissues and K_ATP_ channels are also important in regulating the vascular tone [12]. In rat isolated aorta, cerivastatin-induced a glibenclamide (a K_ATP_ channel blocker)-sensitive aortic relaxation [13] and pravastatin reduced myocardial infact size through opening of mitochondrial K_ATP_ channels in rabbit [14]. However, a recent study reported that simvastatin, but not pravastatin, inhibited pinacidil (a K_ATP_ channel opener)-induced relaxation of pig’s isolated coronary arteries suggesting that different statins have differential effects on K_ATP_ channels of different cells/tissues [40].

Similar to other ion channels, the opening and closing of K_ATP_ channels are modulated by multiple cell signaling mechanisms, such as phosphorylation by protein kinase A (PKA) [15], protein kinase C (PKC) [16] and cGMP-dependent protein kinase (PKG) [17]. In addition, the intracellular ATP level is an essential determinant of K_ATP_ channel gatings. It is well-known that AMP-activated protein kinase (AMPK) serves as a ‘metabolic master regulator’ which is sensitive to changes of intracellular AMP/ATP ratio. Activation of AMPK results in suppression of intracellular energy-consuming pathways and generation of ATP i.e. an increase in cellular ATP level. In mouse isolated pancreatic islets, activation of AMPK by AICAR (an AMPK activator) potentiated insulin secretion by inhibiting K_ATP_ channel openings [18]. Moreover, phenformin (another AMPK activator), inhibited K_ATP_ channel openings in mouse aortic smooth muscle cells [19], highlighting the participation of AMPK activity in K_ATP_ channel gatings in VSMC. Unfortunately, in various *ex vivo* studies (multi-cellular preparations), there is no consensus on the vascular effects mediated by AMPK activation as both contraction and relaxation were observed [20], [21], [22], [23], [24], and the underlying reason(s) for the discrepancy is unknown. Given the fact that statins promoted phosphorylation of AMPK in human and bovine endothelial cells [25], it is tempting to suggest that activation of AMPK by simvastatin could modulate vascular K_ATP_ channel gatings and vascular reactivity.

Therefore, in this study we hypothesize that acute simvastatin could modulate vascular K_ATP_ channel gatings and the simvastatin-mediated effects involve activation of AMPK signaling pathway. Thus, in this study, experiments were designed to evaluate the effects of acute simvastatin on vascular K_ATP_ channel gatings of pig’s coronary artery, and the participation of AMPK activation.

## Materials and Methods

### Animal and Human Ethics Statements

This investigation conformed to the Guide for the Care and Use of Laboratory Animals published by the US National Institute of Health (NIH Publication No. 85–23, revised 1996). The protocol was approved by the Animal Ethics Committee of the Chinese University of Hong Kong (Approval Number: 10/003/DRG). Permission prior to the collection of fresh pig’s heart for research purposes was obtained from Sheung Shui Slaughterhouse (Hong Kong).

Fresh human left internal mammary arteries were the leftover obtained from patients with cardiovascular diseases undergoing coronary artery bypass grafting (CABG) procedures, and the use of human tissues for research purposes was approved by the Human Research Ethics Committee of the Chinese University of Hong Kong (CREC Ref. No. 2006.313). Written consents were obtained, prior to surgery, from patients voluntarily involved for the usage of tissues solely for research purposes. Patients had read and understood the patient information document provided, and the aims and methods of this study had been fully explained to them. Patients involved had given written informed consent (as outlined in PLOS consent form) to authors of this manuscript for publication of these data.

### Isometric Tension Measurement

Fresh hearts were obtained from pigs (∼35 kg) that were slaughtered in the morning of the experiment at a local slaughterhouse. The heart was immediately immersed in an ice-cold physiological salt solution. Segment of the left anterior descending (LAD) coronary artery (tertiary branch, O.D. ∼500–800 µm) was dissected within an hour after the animal was slaughtered.

Fresh human left internal mammary arteries were bathed in an ice-cold physiological salt solution before transported to the laboratory from the operation theatre of the Prince of Wales Hospital (Hong Kong) within an hour. Fat and connective tissues were carefully removed under the dissecting stereo-microscope.

Arterial rings (porcine coronary artery and human internal mammary artery) (endothelium was removed using a blunted watch-maker forceps) were bathed in a 5-ml thermo-regulated wire myograph contained physiological salt solution with the composition (mM): NaCl 118.3, KCl 4.6, MgSO_4_ 1.2, NaHCO_3_ 25, KH_2_PO_4_ 1.2, CaCl_2_ 2.5, and glucose 11 (bubbled with 16%O_2_/5%CO_2_ balanced with N_2_, *p*O_2_ = ∼100 mmHg). Rings (1 mm in length) were equilibrated under resting tension of 1 g [26], [27] using two stainless steel wires (diameter ∼100 µm), in the bath solution for 90 min. Resting tension was re-adjusted, if necessary, before commencing the experiments. The reason for choosing these tissues in this study is because previous reports demonstrated the existence of K_ATP_ channels in these vascular tissues [28], [29].

### Enzymatic Dissociation of Myocytes

Porcine left anterior descending coronary artery myocytes and human left internal mammary artery myocytes were dissociated using collagenase and protease, as reported [3], [13] for conventional whole-cell patch-clamp electrophysiology experiments.

### Electrophysiological Measurement of K_ATP_ Gatings

Conventional whole-cell, patch-clamp experiments were performed at room temperature (∼ 22°C) using single-cell, voltage-clamp techniques (Axopatch 200B amplifier and Digidata 1200 A/D interface) (Axon Instruments, USA) with recording patch pipettes of 2–4 MΩ (when filled with internal pipette solution). Whole-cell recording configurations were used so as to maintain a “pre-determined concentration” of ATP (i.e. 1 mM) inside all the cells used during the recording of K_ATP_ channels for a fair/accurate comparison of K_ATP_ channel gatings of different cells (pig coronary artery and human internal mammary artery) in response to drug challenges. In addition, this mode of recording offers the convenience of a rapid delivery of drugs (e.g. simvastatin Na^+^, okadaic acid and rottlerin) into the cytosol of cells.

The external bath solution contained (mM): KCl 10, potassium gluconate 135, EGTA 5, glucose 5 and HEPES 10 (pH = 7.4). The internal pipette solution contained (mM): KCl 10, potassium gluconate 133, EGTA 5, glucose 5, K_2_ATP 1, NaADP 0.5, MgCl_2_ 1 and HEPES 10 (pH = 7.4) [15]. The cell was held at 0 mV, and pulse voltages from –100 to +40 mV with a 20-mV increment (with pulse duration of 1 s, stimulated at 0.1 Hz) were applied. Current records were low-pass filtered, digitized and stored on computer hard-disk for later analysis using the Clampfit 9 softwares (Axon Instruments, USA).

### Confocal Laser Scanning Microscopy

Porcine coronary artery myocytes were incubated with Fluo-4/AM (5 µM in 0.05% DMSO) (60 min, 37°C) in HEPES buffer (mM): NaCl 140, KCl 5, MgCl_2_ 1, CaCl_2_ 1, glucose 10, and HEPES 10 (pH 7.4). After washing, myocytes were imaged using an Eclipse CL Plus Confocal Microscope System (Nikon, Japan) with an excitation at 488 nm and a band-pass filter at 515/530 nm. Fluorescence changes of myocytes (at room temperature) in response to drugs (simvastatin (10 µM) and AICAR (1 mM), with and without ryanodine (100 µM)) were acquired at 15-s intervals. Images were recorded and analyzed by software EZ-C1 3.5 (Nikon, Japan).

### Measurement of [Glucose]_o_ Uptake

[^3^H]-2-Deoxy-glucose uptake into porcine coronary artery myocytes was determined using previously described protocols with minor modifications [30]. All experiments were performed in HEPES-buffered Ringer’s solution containing (mM): NaCl 135; KCl 5; NaH_2_PO_4_ 3.33; Na_2_HPO_4_ 0.83; CaCl_2_ 1.0; MgCl_2_ 1.0 and HEPES 5 (pH 7.4). Confluent monolayer of cultured porcine coronary artery myocytes in 24-well plates were washed three times in Ringer’s solution. [^3^H]-2-Deoxy-glucose (10 µM, 4 µCi/ml) was added to each well and incubated for 30 min (37°C). The plates were then washed three times rapidly with ice-cold phosphate-buffered saline before cells were solubilized in 0.5 ml of Triton X-100 (5% vol./vol.). To determine non-specific uptake of [^3^H]-2-deoxy-glucose, cells were incubated in buffer containing [^3^H]-2-deoxy-glucose in the presence of cytochalasin B (50 µM) and phloretin (100 µM). The radioactivity was measured using a β-scintillation counter. The protein content was determined spectrophotometrically using the bicinchoninic acid assay (Pierce Biochemicals, USA).

### Determination of Cellular ATP Contents

ATP was extracted from cultured porcine coronary artery myocytes (before and after drug treatments) by trichloroacetic acid (final concentration, 0.5% vol./vol.). Trichloroacetic acid in the sample was then neutralized and diluted to a final concentration of 0.1% by adding Tris-acetate buffer (pH 7.75). The ATP content was analyzed using the ATP-dependent luciferin-luciferase bioluminescence assay (ENLITEN® ATP assay system, Promega, USA).

### Western Immunoblots

Isolated porcine coronary artery and human internal mammary artery (both were endothelium denuded) were homogenized in the presence of protease inhibitors (Roche, USA) to obtain extracts of proteins. Protein concentrations were determined using BCA™ protein assay kit (Pierce, USA). Samples (40 µg of protein per lane) were loaded onto a 10% SDS-polyacrylamide electrophoresis gel. After electrophoresis (90 V, 120 min), the separated proteins were transferred (12 mA, 45 min) to polyvinylidene difluoride membrane (Bio-Rad, USA). Non-specific sites were blocked with 5% non-fat dry milk (Bio-Rad, USA) for 120 min, and the blots were then incubated with individual type of antibody: anti-HMG CoA reductase, 1∶1,000 (Upstate Biotechnology, USA); anti-p-HMG CoA reductase-Ser^871^, 1∶1,000 (Kinasource, UK); anti-CYP450 3A4, 1∶1,000 (Affinity Bioreagent, USA); anti-PP2A, 1∶1,000 (Upstate Biotechnology, USA); anti-p-PP2A-Tyr^307^, 1∶1,000 (Upstate Biotechnology, USA); anti-AMPK, 1∶1,000 (Upstate Biotechnology, USA), anti-p-AMPKα-Thr^172^, 1∶1,000 (Upstate Biotechnology, USA); anti-LKB1, 1∶1,000 (Upstate Biotechnology, USA) and anti-p-LKB1-Ser^428^, 1∶1,000 (Upstate Biotechnology, USA) overnight at 4°C. Anti-mouse HRP conjugated IgG, 1∶1,000 (Bio-Rad, USA) or anti-rabbit HRP conjugated IgG, 1∶1,000 (Bio-Rad, USA) was used to detect the binding of the corresponding antibody. Membranes were stripped and re-blotted with anti-β-actin antibody, 1∶10,000 (Sigma-Aldrich, USA) to verify an equal loading of protein in each lane. The protein expression was detected with ImmueStar Reagent (Bio-Rad, USA) and quantified using Scion Image analysis programme (Scion Image Ltd., USA).

### Chemicals

Simvastatin, cromakalim, glibenclamide, AICAR, compound C, rottlerin, ryanodine, caffeine, U46619, KB R-7953 mesylate, H89 dihydrochloride, okadaic acid, KN93 and KN92 were purchased from Tocris Biosciences (UK). Simvastatin Na^+^ (50 mmol/L) was prepared from simvastatin using NaOH (in ethanol), as suggested by the manufacturer (Tocris Biosciences, UK). Nifedipine, D-mannitol, pinacidil, ouabain, phloridzin, 5-(N-ethyl-N-isopropyl) amiloride (EIPA), N-methyl-D-glucamine chloride and ketoconazole were obtained from Sigma-Aldrich (USA).

### Statistical Analysis

All data were obtained from at least 6 independent experiments. Statistical analysis was performed using Student’s *t* test or ANOVA (one-way or two-way), where appropriate. A *P* value of <0.05 was considered significant. Data are expressed as mean ± S.EM.

## Results

### Biochemical Existence of HMG-CoA Reductase and the Effects of Simvastatin and Simvastatin Na^+^ on HMG-CoA Reductase Expression

**Figure 1:**
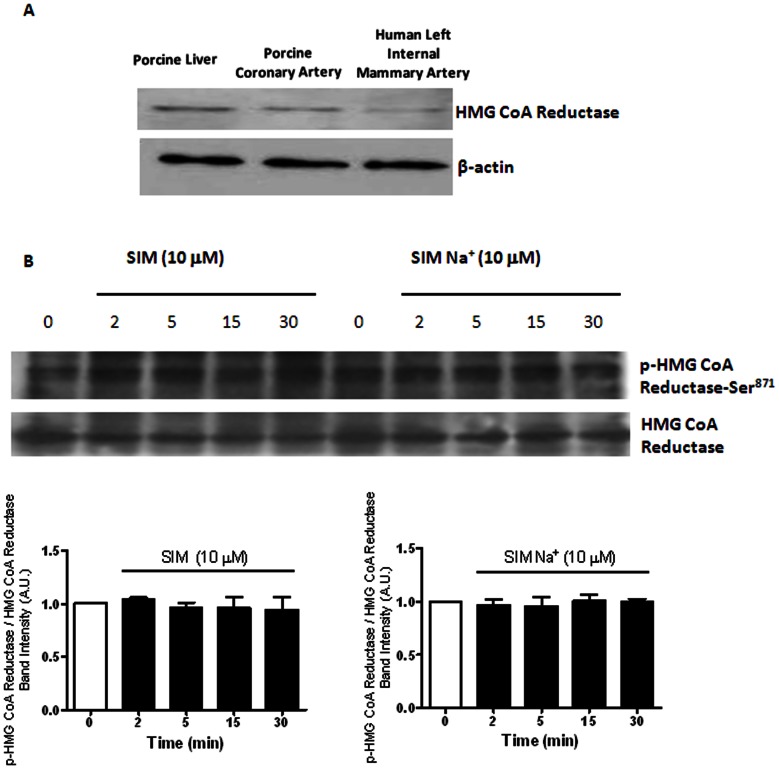
Biochemical existence of HMG-CoA reductase, and the effects of simvastatin and simvastatin Na^+^ on the protein expression of HMG-CoA reductase and p-HMG-CoA reductase. (A) Biochemical existence of HMG-CoA reductase in porcine liver, porcine coronary artery (endothelium-denuded) and human left internal mammary artery (endothelium-denuded). Beta actin was used as loading control. (B) Effects of simvastatin (SIM) (10 μM) and simvastatin Na^+^ (SIM Na^+^) (10 μM) (incubation, 2 to 30 min) on the protein expression of p-HMG-CoA reductase-Ser^871^ and HMG-CoA reductase of porcine coronary artery.

The biochemical existence of HMG CoA reductase was determined in both human isolated left internal mammary artery and porcine isolated coronary artery. Porcine liver served as the positive control. Western blot results confirmed the biochemical existence of HMG-CoA reductase in both human and porcine vascular preparations. Beta actin was used as a loading control (Figure 1A).

We then investigated the effects of simvastatin and simvastatin Na^+^ in inhibiting HMG CoA reductase activity (i.e. phosphorylation of HMG CoA reductase) [29]. Neither simvastatin nor simvastatin Na^+^ (10 µM, incubation ≤ 30 min) altered the protein expression of p-HMG-CoA reductase-Ser^871^ and HMG-CoA reductase in porcine isolated coronary artery (Figure 1B).

### Effects of Simvastatin on K_ATP_ Channel Opener-induced Relaxation

**Figure 2:**
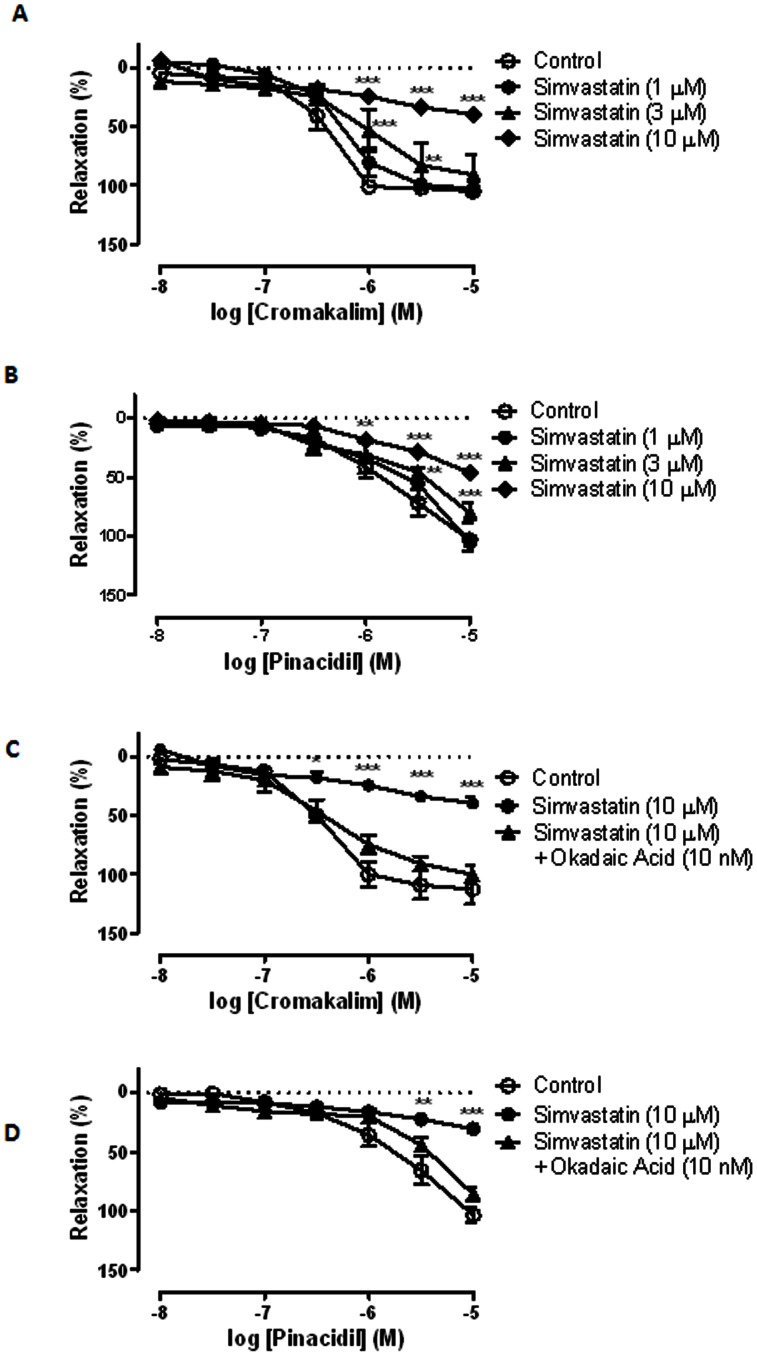
Effects of simvastatin on K_ATP_ channel openers-induced vasorelaxation. (A) Effect of simvastatin (1, 3 and 10 μM) (n = 6 to 8) on cromakalim-induced relaxation of U46619 (10 nM) pre-contracted porcine coronary artery (endothelium-denuded). (B) Effect of simvastatin (1, 3 and 10 μM) (n = 6 to 8) on pinacidil-induced relaxation of U46619 (10 nM) pre-contracted porcine coronary artery (endothelium-denuded). (C) Effect of okadaic acid (10 nM) (n = 6 to 8) on simvastatin-inhibited cromakalim-induced relaxation of U46619 (10 nM) pre-contracted porcine coronary artery (endothelium-denuded). (D) Effect of okadaic acid (10 nM) (n = 6 to 8) on simvastatin-inhibited pinacidil-induced relaxation of U46619 (10 nM) pre-contracted porcine coronary artery (endothelium-denuded). *P<0.05, **P<0.01 and ***P<0.001 compared to controls.

To evaluate the involvement of K_ATP_ channels, effects of simvastatin on K_ATP_ channel opener-mediated vascular relaxation were examined. Cromakalim and pinacidil (both are K_ATP_ channel openers) (10 nM to 10 µM) caused a glibenclamide (1 and 3 µM)-sensitive relaxation of U46619 (10 nM) pre-constricted coronary artery (endothelium-denuded) relaxation in a concentration-dependent manner (data not shown). Glibenclamide alone did not alter the basal tension and U46619-induced contraction. Simvastatin (3 and 10 µM), but not simvastatin Na^+^ (1, 3 and 10 µM), significantly attenuated cromakalim- and pinacidil-induced relaxation (Figure 2A and B). Neither simvastatin nor simvastatin Na^+^ altered the basal tension of the preparation.

Okadaic acid (a potent PP2A inhibitor) was used to elucidate the involvement of PP2A in simvastatin-suppressed cromoakalim- and pinacidil-inudced relaxation. Okadaic acid (10 nM) eradicated simvastatin (10 µM)-induced inhibition of cromakalim- and pinacidil-induced relaxation (n = 6) (Figure 2C and D). Okadaic acid (10 nM) alone did not modify cromakalim-induced relaxation.

### Effects Simvastatin on K_ATP_ Openings

**Figure 3:**
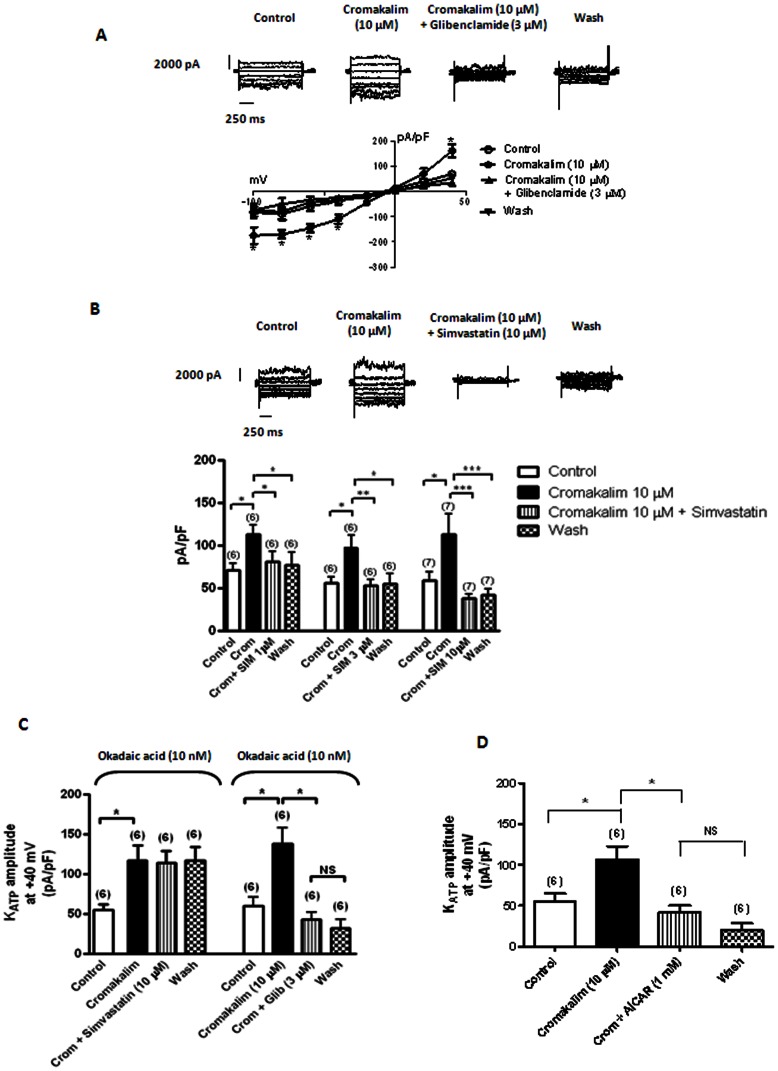
Effects of simvastatin on K_ATP_ channel openings. (A) Effects of cromakalim (Crom., 10 μM) on whole-cell K_ATP_ channel openings of single human internal mammary artery myocytes in the presence of glibenclamide (Glib., 3 μM) (n = 5 to 6). (B) Effects of cromakalim (Crom., 10 μM) on whole-cell K_ATP_ channel openings of single human internal mammary artery myocytes with and without simvastatin (1, 3 and 10 μM). (C) Effects of simvastatin (10 μM) and glibenclamide (Glib., 3 μM) on cromakalim (Crom., 10 μM)-induced whole-cell K_ATP_ channel openings of single porcine artery myocytes (in the presence of okadaic acid, 10 nM). Number of cells studied is indicated in parenthesis. *P<0.05, **P<0.01 and ***P<0.001 compared to controls. (D) Effects of cromakalim (Crom., 10 μM) on whole-cell K_ATP_ channel openings of single porcine coronary artery myocytes in the presence of AICAR (1 mM). Number of cells studied is indicated in parenthesis. *P<0.05, **P<0.01 and ***P<0.001 compared to controls.

In order to get a better understanding on the modulation of K_ATP_ channels gatings by simvastatin, experiments were performed in single vascular myocytes. Cromakalim (10 µM) (Figure 3A) and pinacidil (10 µM) (data not shown) significantly enhanced the recorded outward K^+^ current amplitude which is inhibited by glibenclamide (a K_ATP_ channel blocker), indicating that the recorded K^+^ current is the genuine K_ATP_ current.

In human internal mammary artery myocytes, neither simvastatin (1, 3 and 10 µM) nor simvastatin Na^+^ (1, 3 and 10 µM) altered the basal K_ATP_ channel gatings (data not shown). Interestingly, simvastatin caused a concentration-dependent inhibition of cromakalim (10 µM)-induced K_ATP_ channel opening, with no apparent recovery after washout (Figure 3B). However, simvastatin Na^+^ (10 µM, applied either in external bath solution or included in the pipette solution) did not alter cromakalim (10 µM)-induced K_ATP_ opening (data not shown).

Due to the irregular/limited supply of human left internal mammary artery for research purposes, the following experiments were performed using porcine coronary artery myocytes. All drugs/inhibitors were tested against both cromakalim- and pinacidil-induced K_ATP_ opening, however only representative figures of drug modulation of cromakalim-mediated responses were illustrated in the Figures.

Okadaic acid (a potent PP2A inhibitor) was used to examine the involvement of PP2A in simvastatin-mediated suppression of cromakalim- and pinacidil-induced K_ATP_ opening. Okadaic acid (10 nM) did not alter the basal K_ATP_ openings, and cromakalim (10 µM)- and pinacidil (10 µM)-induced K_ATP_ openings. However, okadaic acid (10 nM, in the pipette solution) significantly attenuated simvastatin (10 µM)-mediated suppression of cromakalim (10 µM)- and pinacidil (10 µM)-induced K_ATP_ openings (Figure 3C). In contrast, okadaic acid failed to alter glibenclamide (3 µM)-mediated inhibition of cromakalim- and pinacidil-induced K_ATP_ openings (Figure 3C).

The involvement of AMPK on cromakalim- and pinacidil-induced K_ATP_ channel openings was examined. Similar to simvastatin (10 µM), AICAR (1 mM, an AMPK activator) attenuated cromakalim- and pinacidil-induced K_ATP_ channel openings (Figure 3D). However, AICAR (1 mM) did not alter the basal K_ATP_ amplitude (data not shown).

### Effects of Simvastatin on AMPK and PP2A Phosphorylation

**Figure 4:**
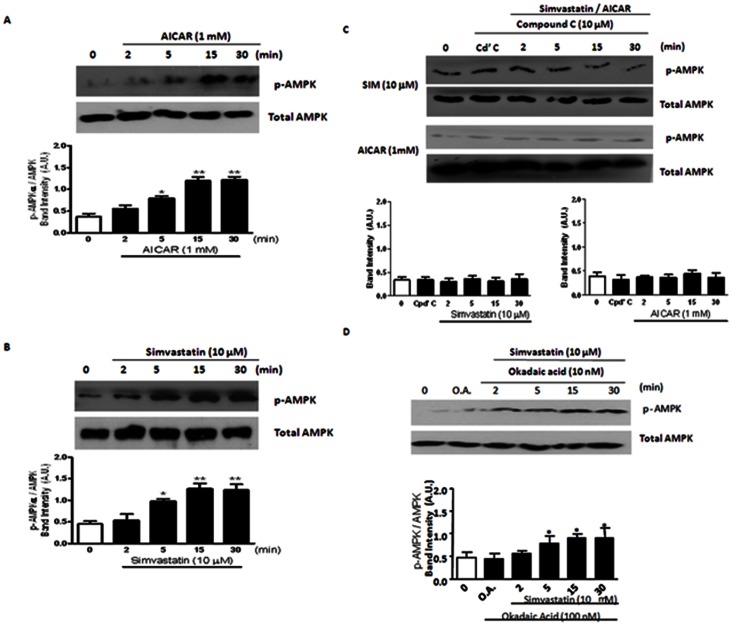
Effects of simvastatin on AMPK activation in porcine coronary artery. (A) Effects of AICAR (1 mM) on the protein expression of p-AMPK/total AMPK in porcine coronary artery. *P<0.05 and **P<0.01 compared to controls (i.e. time 0). (B) Effects of simvastatin (10 μM) on the protein expression of p-AMPK/total AMPK in porcine coronary artery. *P<0.05 and **P<0.01 compared to controls (i.e. time 0). (C) Effect of compound C (10 μM) on simvastatin- and AICAR-induced AMPK activation (p-AMPK/total AMPK) in porcine coronary artery. *P<0.05 and **P<0.01 compared to controls (i.e. time 0). (D) Effect of okadaic acid (10 nM) on simvastatin-induced AMPK activation (p-AMPK/total AMPK) in porcine coronary artery. *P<0.05 and **P<0.01 compared to controls (i.e. time 0).

To strengthen our hypothesis on the participation of AMPK activation on mediating simvastatin-induced responses, we evaluated AMPK activity using Western blots. Activity of AMPK is represented by p-AMPKα-Thr^172^/total AMPK, as previously demonstrated [31]. AICAR (1 mM) and simvastatin (10 µM) caused a time-dependent (2–30 min) increase of AMPK activation (i.e. increased p-AMPKα-Thr^172^ expression) (Figure 4A and B). These responses were sensitive to Compound C (an AMPK inhibitor) (1 µM; 30 min) (Figure 4C). Okadaic acid (10 nM, 30 min) (Figure 4D) did not alter simvastatin (10 µM)-mediated increase of AMPK activity.

**Figure 5:**
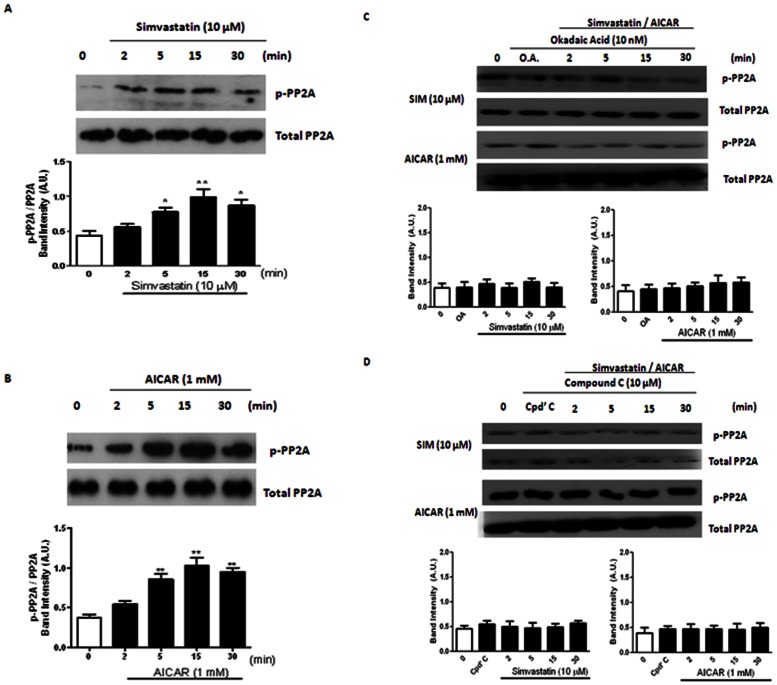
Effects of simvastatin on PP2A activation in porcine coronary artery. (A) Effects of simvastatin (10 μM) on the protein expression of p-PP2A/total PP2A in porcine coronary artery. *P<0.05 and **P<0.01 compared to controls (i.e. time 0). (B) Effects of AICAR (1 mM) on the protein expression of p-PP2A/total PP2A in porcine coronary artery. *P<0.05 and **P<0.01 compared to controls (i.e. time 0). (C) Effect of okadaic acid (O.A., 10 nM) on simvastatin- and AICAR-induced protein expression of p-PP2A/total PP2A in porcine coronary artery. *P<0.05 and **P<0.01 compared to controls (i.e. time 0). (D) Effect of compound C (10 μM) on simvastatin- and AICAR protein expression of p-PP2A/total PP2A in porcine coronary artery. *P<0.05 and **P<0.01 compared to controls (i.e. time 0).

The role of PP2A activation (represented by p-PP2A-Tyr^307^/total PP2A), as previously reported [32], in response to simvastatin and AICAR challenges was evaluated using okadaic acid and Compound C. Simvastatin (10 µM) and AICAR (1 mM) elicited a time-dependent (2 to 30 min) increase in p-PP2A-Tyr^307^/total PP2A (i.e. a decreased PP2A activity) (Figure 5A and B). Simvastatin (10 µM)- and AICAR (1 mM)-induced decrease of PP2A activity was eradicated by okadaic acid (10 nM, 30 min) and Compound C (1 µM, 30 min) (Figure 5C and D).

### Role(s) of [Ca^2+^]_o_ and [Ca^2+^]_i_ in Mediating Effects of Simvastatin on AMPK and PP2A Activities

**Figure 6:**
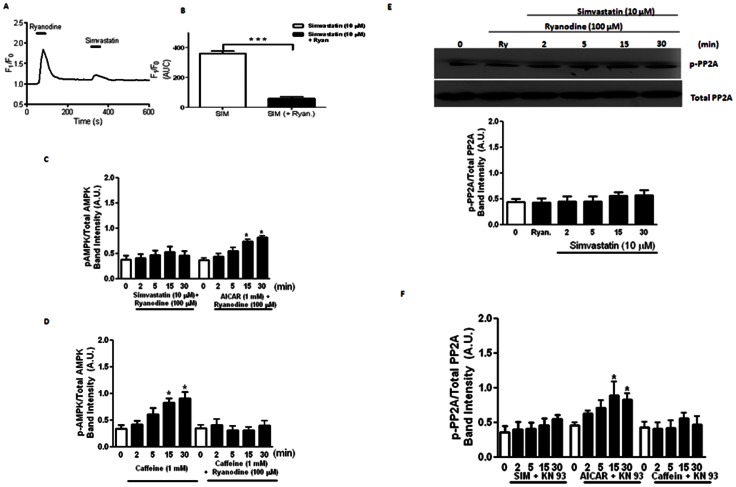
Role(s) of [Ca^2+^]_o_ and [Ca^2+^]_i_ in mediating the effects of simvastatin on AMPK and PP2A activities. (A) Effects of simvastatin (10 μM), with and without ryanodine (100 μM) pre-treatment, on [Ca^2+^]_i_ changes (F_1_/F_0_) of porcine coronary artery myocytes, estimated using Fluo-4/AM with confocal laser scanning microscope. (B) Summary of [Ca^2+^]_i_ changes in response to simvastatin (10 μM) before and after ryanodine (100 μM) challenges. Results are expressed (Area Under Curve, AUC) as mean ± SEM of 13–15 cells (***P<0.001). (C) Summary of the effects of ryanodine (100 μM) on simvastatin (10 μM)- or AICAR (1 mM)-induced protein expression of p-AMPK/total AMPK in porcine coronary artery. *P<0.05 and **P<0.01 compared to controls (i.e. time 0). (D) Summary of the effects of caffeine (1 mM) on the protein expression of p-AMPK/total AMPK in porcine coronary artery, with and without ryanodine (100 μM). *P<0.05 and **P<0.01 compared to controls (i.e. time 0). (E) Effect of ryanodine (100 μM) on simvastatin (10 μM)-induced protein expression of p-PP2A/total PP2A in porcine coronary artery. *P<0.05 and **P<0.01 compared to controls (i.e. time 0). (F) Summary of the effect of simvastatin (10 μM), AICAR (1 mM) and caffeine (1 mM) on the protein expression of p-PP2A/total PP2A, with and without KN93 (10 μM) in porcine coronary artery. *P<0.05 and **P<0.01 compared to controls (i.e. time 0).

Ca^2+^ ions are important in mediating various cellular signaling cascades. The significance of [Ca^2+^]_o_ and [Ca^2+^]_i_ in mediating simvastatin-induced responses was therefore evaluated. Simvastatin (10 µM) caused an increase in [Ca^2+^]_i_ level and contraction of single myocytes (data not shown). In myocytes challenged with ryanodine (100 µM), there was a transient increase in [Ca^2+^]_i_, (plus contraction of single myocytes) and the subsequent application of simvastatin (10 µM) (Figure 6A and B) failed to alter [Ca^2+^]_i_ levels.

To elucidate the role of changes in [Ca^2+^]_i_ in mediating simvastatin-induced AMPK activation as shown above, effects of ryanodine, [Ca^2+^]_o_-free solution, KB R-7953 and nifedipine were examined. Ryanodine (100 µM, 30 min pre-treatment) abolished simvastatin (10 µM)-, but not AICAR (1 mM)-, induced AMPK activation (Figure 6C). Caffeine (1 mM) caused a time-dependent (2 to 30 min) (ryanodine-sensitive) AMPK activation (Figure 6D). Ryanodine, on its own, did not alter AMPK activation.

Ryanodine pre-treatment (100 µM) abolished simvastatin (10 µM)-, AICAR (1 mM) and caffeine (1 mM)-induced changes of p-PP2A-Tyr^307^/total PP2A (Figure 6E). Moreover, effects of simvastatin (10 µM)- and caffeine (1 mM)-, but not AICAR (1 mM)-, on PP2A activities were abolished by KN 93 (10 µM) (Figure 6F), but not by KN 92 (10 µM) (data not shown). In addition, effects of simvastatin (10 µM) and AICAR (1 mM) on AMPK and PP2A activities were not modified by [Ca^2+^]_o_-free solution (with EGTA, 2 mM), KB R-7953 (10 µM)- or nifedipine (10 µM)-containing solutions (data not shown).

### Effects Simvastatin and AICAR on [Glucose]_o_ Uptake

**Figure 7:**
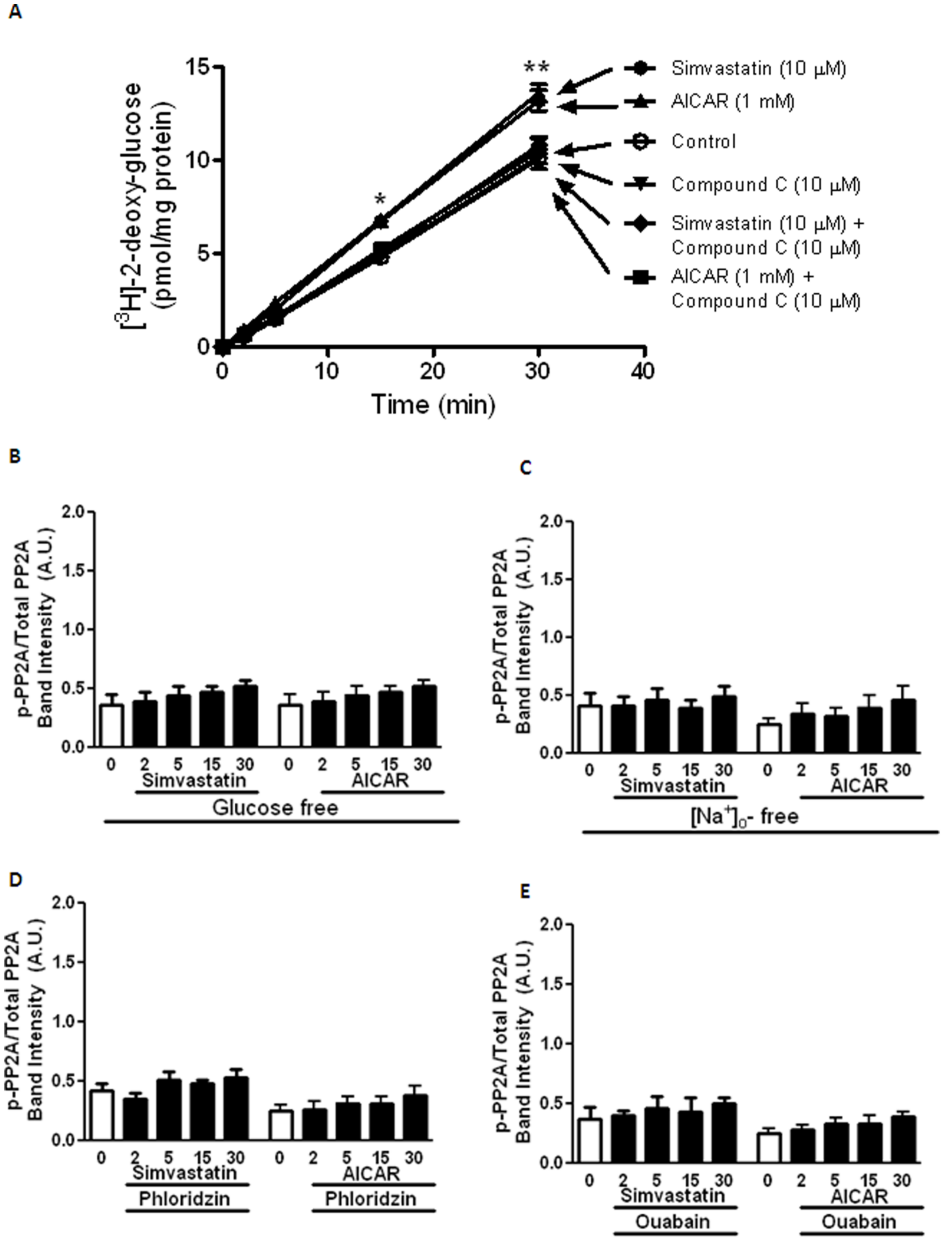
Effects of simvastatin and AICAR on [Glucose]_o_ uptake and the role(s) of [glucose]_o_ and [Na^+^]_o_ in mediating simvastatin effects on AMPK and PP2A activities. (A) Effects of simvastatin (10 μM) and AICAR (1 mM) on [^3^H]-2-deoxy-glucose uptake, with and without compound C (10 μM), of porcine coronary artery myocytes (n = 6 for each treatment). *P<0.05 and **P<0.01 compared to controls. Summary of the effect of simvastatin and AICAR on the protein expression of p-PP2A/total PP2A in (B) [glucose]_o_-free, (C) [Na^+^]_o_-free, (D) with phloridzin (1 mM) and (E) with ouabain (10 μM) in porcine coronary artery. *P<0.05 and **P<0.01 compared to controls (i.e. time 0).

In order to establish the role of [glucose]_o_, effects of simvastatin and AICAR on [glucose]_o_ uptake was determined. Simvastatin (10 µM) and AICAR (1 mM) caused a significant increase in [^3^H]-2-deoxy-glucose uptake into coronary artery myocytes, and the “enhanced” [glucose]_o_ uptake was eradicated by Compound C (10 µM) (Figure 7A).

### The Role(s) of [Glucose]_o_ and [Na^+^]_o_ in Mediating Simvastatin Effects on AMPK and PP2A Activities

After the confirmation of the essential role of [glucose]_o_ as mentioned above, identification of the [glucose]_o_ uptake transporter involved was performed. [Glucose]_o_-free solution (osmotic balanced with D-mannitol), phloridzin (1 mM, a Na^+^/glucose co-transporter-1 (SGLT-1) blocker), ouabain (10 µM, a Na^+^/K^+^ ATPase inhibitor), 5-(N-ethyl-N-isopropyl) amiloride (EIPA, 10 µM) (a Na^+^/H^+^ exchanger-1 blocker) and [Na^+^]_o_-free (replaced with N-methyl-D-glucamine) solution failed to alter simvastatin (10 µM)- and AICAR (1 mM)-mediated increase of p-AMPKα-Thr^172^ expression (data not shown). In contrast, simvastatin (10 µM)- and AICAR (1 mM)-mediated increase of p-PP2A-Tyr^307^/total PP2A (i.e. PP2A inhibition) was eradicated in [glucose]_o_-free (Figure 7B) or [Na^+^]_o_-free conditions (Figure 7C), and with phloridzin (1 mM)- (Figure 7D) or ouabain (10 µM)-containing solutions (Figure 7E). However, EIPA (10 µM) did not modify simvastatin (10 µM)- and AICAR (1 mM)-mediated changes of p-PP2A-Tyr^307^/total PP2A (data not shown).

### Effects of Simvastatin on [ATP]_i_ Levels and LKB1 Activation

**Figure 8:**
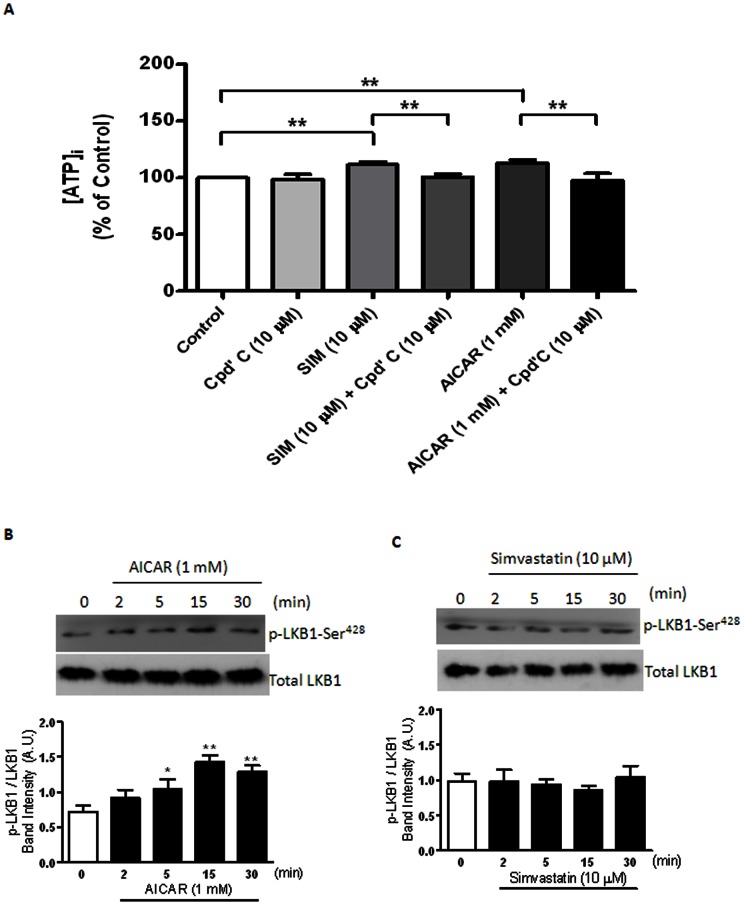
Effects of simvastatin on [ATP]_i_ levels and LKB1 activation. (A) Summary of the effects of simvastatin (10 μM) and AICAR (1 mM) on [ATP]_i_ level with and without compound C (10 μM) of porcine coronary artery myocytes (n = 6 for each treatment). *P<0.05 and **P<0.01 compared to controls. (B) Effect of AICAR (1 mM, n = 4) on the protein expression of p-LKB1/total LKB1 in porcine coronary artery. *P<0.05 and **P<0.01 compared to controls (i.e. time 0). (C) Effect of simvastatin (10 μM, n = 4) on the protein expression of p-LKB1/total LKB1 in porcine coronary artery. *P<0.05 and **P<0.01 compared to controls (i.e. time 0).

To confirm the generation of [ATP]_i_ after [glucose]_o_ uptake induced by simvastatin, cellular ATP level was estimated in response to drug challenges. AICAR caused a Compound C (10 µM)-sensitive increase in cellular ATP level of the arterial myocytes (Figure 8A) and a time-dependent (2 to 30 min) increase in LKB1 activity (i.e. an increase in p-LKB1-Ser^428^/total LKB1 [33]) (Figure 8B). However, simvastatin increased intracellular ATP level of the arterial myocytes with no apparent change in p-LKB1/total LKB1 (Figure 8C).

### Participation of Cytochrome P450 3A4

**Figure 9:**
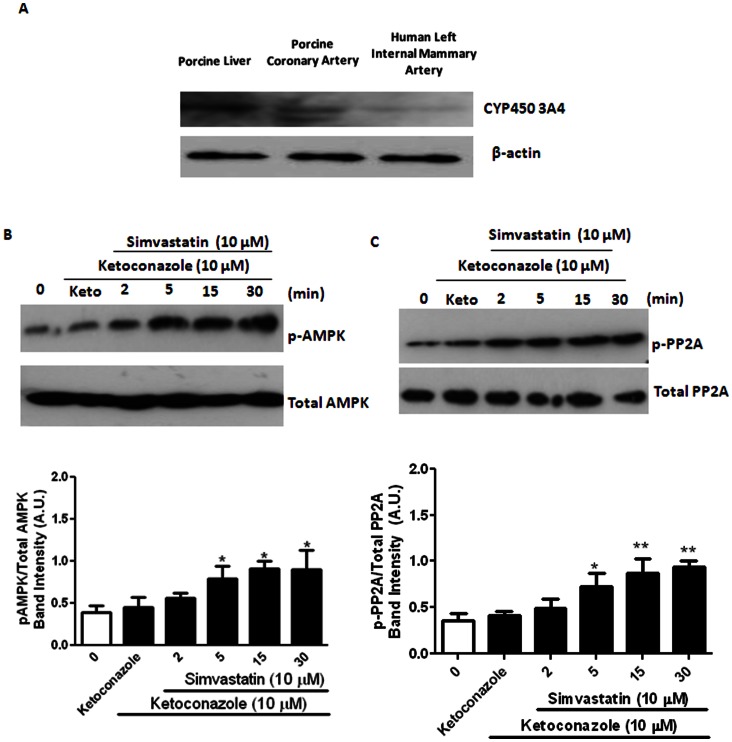
Participation of cytochrome P450 3A4. (A) Biochemical existence of cytochrome 450 (CYP450 3A4) in porcine liver, porcine coronary artery (endothelium denuded) and human left internal mammary artery (endothelium denuded). Beta actin was used as loading control. (B) Effects of simvastatin on the protein expression of p-AMPK/total AMPK, with ketoconazole (Keto, 10 μM, n = 4), in porcine coronary artery (endothelium denuded). *P<0.05 and **P<0.01 compared to controls (i.e. time 0). (C) Effects of simvastatin on the protein expression of p-PP2A/total PP2A, with ketoconazole (Keto, 10 μM, n = 4), in porcine coronary artery (endothelium denuded). *P<0.05 and **P<0.01 compared to controls (i.e. time 0).

To elucidate the importance of cytochrome P450 (CYP450)-mediated drug metabolism in mediating simvastatin-induced responses, effects of CYP450 3A4 inhibitor was examined. The biochemical existence of CYP450 3A4 protein was confirmed in porcine coronary artery and human internal mammary artery (Figure 9A). Porcine liver served as the positive control. Ketoconazole (10 µM, a selective CYP450 3A4 inhibitor) failed to modify simvastatin (10 µM)-induced changes of AMPK and PP2A activities (Figure 9B and C).

## Discussion

In this study, acute simvastatin (membrane permeable) suppressed cromakalim- and pinacidil-induced relaxation of U46619 pre-constricted (endothelium-denuded) arteries with no effects on basal tension. In single myocytes of porcine coronary artery and human left internal mammary artery, simvastatin and AICAR inhibited cromakalim- and pinacidil-evoked K_ATP_ channel openings with no apparent effect on basal K_ATP_ channel gatings. Thus, a prerequisite opening of K_ATP_ channels (by two structurally different K_ATP_ channel openers cromakalim and pinacidil) is necessary for simvastatin and AICAR to demonstrate K_ATP_ channel blocking properties. However, simvastatin Na^+^ (membrane impermeable) did not alter the cromakalim−/pinacidil-induced relaxation and the K_ATP_ channel openings. Therefore, these results suggest that the lipophilic property of simvastatin is essential [3], [34].

Acute application of HMG-CoA reductase inhibitors (pravastatin, atorvastatin and cerivastatin) elicited an endothelium-dependent relaxation of pre-constricted rat isolated aorta, and cerivastatin-induced relaxation was attenuated by glibenclamide and ouabain [13]. Activation of cardiac K_ATP_ channels by statins has been reported [14], [35], [36]. However, a recent study [37] and our current study demonstrated that simvastatin inhibits pinacidil-induced relaxation in porcine isolated coronary artery. Furthermore, our result illustrate that simvastatin consistently suppressed, instead of enhanced, cromakalim- and pinacidil-induced K_ATP_ channel openings of arterial myocytes of pig coronary artery and human left internal mammary artery. Taken together, these results clearly illustrate that simvastatin could alter vasodilatation via the inhibition of K_ATP_ channels.

AMPK (formerly termed HMG-CoA reductase kinase) is activated by an increase in [AMP/ATP]_i_ ratio and a rise in [Ca^2+^]_i_ which signals an increase in energy demands [38], [39]. Once activated, AMPK decreases ATP consumption and/or stimulates ATP production (e.g. via oxidative phosphorylation) and the [ATP]_i_ level is thus restored [40]. In human umbilical vein endothelial cells (HUVECs), atorvastatin activated AMPK [25] whereas in mouse pancreatic islets β-cells, AICAR (an AMPK activator) inhibited K_ATP_ openings [41]. Acute application of simvastatin significantly suppressed vasoconstriction of rat mesenteric resistance arteries via an AMPKα-phosphorylation-dependent mechanism [42]. In addition, AICAR activates AMPK via an increased phosphorylation, and phosphorylation (i.e. inactivation) of a known target for AMPK i.e. HMG-CoA reductase occurred [18], [43], [44]. However, in our study neither simvastatin nor simvastatin Na^+^ (incubation ≤ 30 min) altered the expression of HMG-CoA reductase and p-HMG-CoA reductase-Ser^871^ (the inactivated isoform of HMG-CoA reductase) suggesting that AICAR and simvastatin are acting through different cellular mechanisms (see below). In rat’s liver, AMPK is associated with HMG-CoA reductase [45]. In our study, simvastatin and AICAR consistently increased p-AMPKα-Thr^172^ expression (i.e. activation) [44], and suppressed cromakalim- and pinacidil-induced K_ATP_ channel openings. Taken together, our results illustrate that acute simvastatin inhibits vascular K_ATP_ channel openings probably via AMPKα-Thr^172^ phosphorylation (i.e. activation).

Okadaic acid (a potent PP2A inhibitor), but not rotterlin (a PKC-δ inhibitor) or H89 (a PKA inhibitor), reversed the inhibitory effects of simvastatin on cromakalim- and pinacidil-mediated K_ATP_ channel openings strengthen our conclusion on the participation of PP2A. In contrast to cultured bovine aortic endothelial cells (BAECs) [46], simvastatin- and AICAR-mediated increase in PP2A-Tyr^307^ phosphorylation was abolished by Compound C (an AMPK inhibitor) illustrating that PP2A is phosphorylated (i.e. inactivated) in an “AMPKα-dependent” manner, and PP2A phosphorylation occurs at a site downstream of AMPKα activation/phosphorylation. In addition, AMPK can be activated via the LKB1 (the upstream serine/threonine kinase of AMPK) cascade [47]. In cultured BAECs, simvastatin (10 µM) increased AMPKα-Thr^172^ phosphorylation via LKB1-Ser^428^ phosphorylation [5], [8]. However, in our study, simvastatin (unlike AICAR) did not cause LKB1-Ser^428^ phosphorylation of porcine coronary artery suggesting that simvastatin-induced AMPKα-Thr^172^ phosphorylation is mediated via a LKB1-Ser^428^ phosphorylation-independent pathway.

Apart from LKB1, AMPK is activated/phosphorylated by Ca^2+^/calmodulin-dependent kinase kinase (CaMKK) [48]. In our study, caffeine- and simvastatin-induced AMPKα-Thr^172^ phosphorylation was abolished by KN93 (a selective Ca^2+^/calmodulin-dependent protein kinase II (CaMK II) inhibitor) illustrating the participation of Ca^2+^/CaMK II. In contrast, AICAR-induced AMPKα-Thr^172^ phosphorylation was not affected by KN93 suggesting that AICAR-mediated AMPKα-Thr^172^ phosphorylation is CaMK II-independent [48]. In fact, our results illustrate that AICAR increased p-LKB1-Ser^428^ expression suggesting that AICAR phosphorylates AMPKα-Thr^172^ via the LKB1 pathway [49].

Simvastatin inhibited Ca^2+^ release from intracellular stores of smooth muscle cells [50], [51]. As mentioned above, caffeine- and simvastatin-induced AMPKα-Thr^172^ phosphorylation involved CaMK II activation which is a Ca^2+^-dependent process. Similar to rat aorta [52] and cultured BAECs [53], in our study simvastatin caused a ryanodine-sensitive [Ca^2+^]_i_ increase in porcine coronary artery myocytes. Apart from Ca^2+^ release from intracellular stores, the [Ca^2+^]_i_ level is also modified by [Ca^2+^]_o_ influx [54]. However, our results reveal that [Ca^2+^]_o_-free conditions did not modify simvastatin-induced p-AMPKα-Thr^172^ expression. Neither nifedipine (a L-type Ca^2+^ channel blocker) nor KB R-7953 (a reverse-mode Na^+^/Ca^2+^ exchanger blocker) altered simvastatin-induced AMPKα-Thr^172^ phosphorylation. Taken together, the increased p-AMPKα-Thr^172^ expression is solely dependent on ryanodine-sensitive [Ca^2+^]_i_ release which is probably related to the distinct physiological location of HMG-CoA reductase (i.e. sarco/endoplasmic reticulum) [1].

Activation of AMPK causes [glucose]_o_ uptake [55], and in yeast AMPK activation is regulated by PP2A in a [glucose]_o_-dependent manner [56]. Although simvastatin and AICAR caused a Compound C-sensitive increase in [^3^H]-2-deoxy-glucose uptake into the myocytes, simvastatin- and AICAR-induced p-AMPKα-Thr^172^ expression was [glucose]_o_−/[Na^+^]_o_-independent. Hence, ryanodine-sensitive Ca^2+^ release and activation of CaMK II, but not [glucose]_o_ uptake, are essential initial steps necessary for AMPKα-Thr^172^ phosphorylation upon simvastatin challenge. Nonetheless, our results demonstrate that simvastatin and AICAR caused an increase in [ATP]_i_ levels. The elevated [ATP]_i_ contributed to the closure of vascular K_ATP_ channels [57] as well as providing phosphate groups necessary for protein (e.g. PP2A-Tyr^307^) phosphorylation (see below).

AMPK is regulated negatively by serine/threonine phosphatase(s) e.g. PP2A [46], and in rat’s liver HMG-CoA reductase is associated with PP2A [45]. Our results clearly illustrate that simvastatin-induced PP2A-Tyr^307^ phosphorylation (i.e. inactivation) [58] requires [glucose]_o_. In addition, Na^+^/K^+^-ATPase provides the favorable trans-cellular Na^+^ gradient for [glucose]_o_ uptake via Na^+^-glucose co-transporter (SGLT-1) [59]. Phloridzin (a SGLT-1 inhibitor), [Na^+^]_o_ depletion or ouabain suppressed PP2A-Tyr^307^, but not AMPKα-Thr^172^, phosphorylation illustrating the obligatory role of [glucose]_o_ uptake via SGLT-1 [60] with the participation of Na^+^/K^+^-ATPase for PP2A-Tyr^307^ phosphorylation.

The biochemical existence of cytochrome P450 (CYP450) 3A4 was demonstrated in porcine coronary artery and human left internal mammary artery, and the possible local enzymatic conversion of simvastatin into simvastatin Na^+^ by CYP450 3A4 [61] was considered. However, ketoconazole (a selective CYP450 3A4 inhibitor) failed to modify simvastatin-induced increase in p-AMPKα-Thr^172^ and p-PP2A-Tyr^307^ expression refuted the possibility of local bio-transformation of simvastatin. Thus, our results strengthen the conclusion on the involvement of simvastatin, but not simvastatin Na^+^, in inhibiting vascular K_ATP_ channel openings.

**Figure 10:**
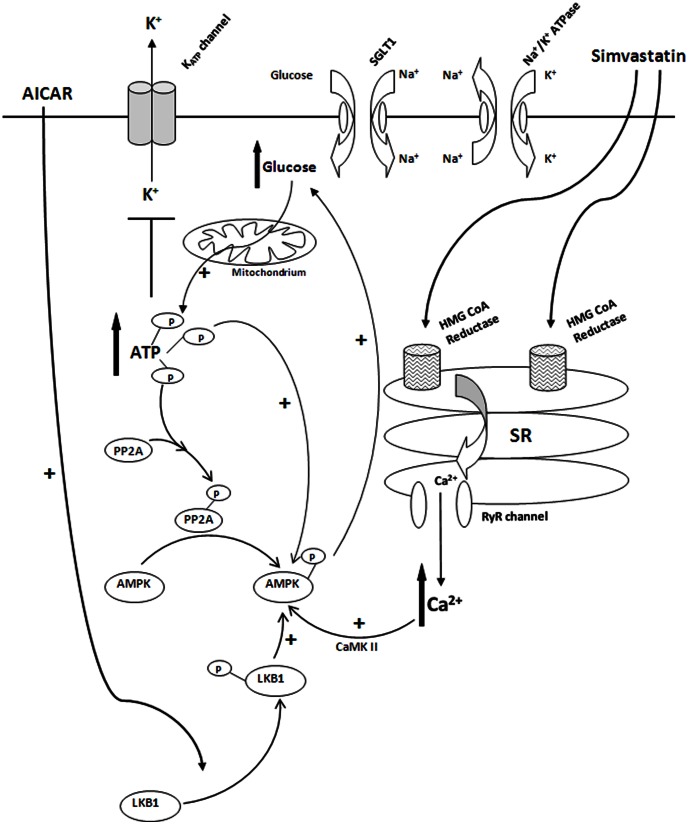
Proposed mechanisms for acute simvastatin-induced closure of K_ATP_ channels of vascular myocytes. Simvastatin (lipophilic) crosses the plasma membrane and reaches the sacroplasmic reticulum (SR) of vascular myocytes. Binding of simvastatin to SR leads to the release of ryanodine (Ryr)-sensitive Ca^2+^ into the cytosol. Elevation of Ca^2+^ activates CaMK II which leads to the subsequent activation (phosphorylation) of AMPKα. Phosphorylation of AMPKα-Thr^172^ causes [glucose]_o_ uptake with the participation of SGLT1 and Na^+^/K^+^ ATPase. Increase in cytosolic [glucose] leads to an elevation of ATP levels via oxidative phosphorylation. Elevation of [ATP]_i_ serves two purposes: (1) closure of vascular K_ATP_ channels, (2) providing phosphate groups for cellular proteins (e.g. PP2A and AMPK) phosphorylation. Phosphorylation of PP2A occurs downstream of AMPK phosphorylation. PP2A phosphorylation results in PP2A inactivation which “releases” AMPK and thus phosphorylation of AMPKα-Thr^172^ resulted. AICAR produces similar effects as simvastatin except the initial step involves LKB1-Ser^428^ phosphorylation.

In conclusion, our results demonstrate that acute simvastatin caused phosphorylation of PP2A-Tyr^307^ and AMPKα-Thr^172^, but not HMG-CoA reductase-Ser^871^, of porcine coronary artery. Ryanodine-sensitive Ca^2+^ stores, but not [Ca^2+^]_o_ entry, play an obligatory role in simvastatin-elicited [Ca^2+^]_i_ increase and initiated the activation of Ca^2+^/CaMK II cascade which are essential for the subsequent AMPKα-Thr^172^ phosphorylation (activation). Activation of AMPKα leads to [glucose]_o_ uptake (and [ATP]_i_ elevation resulted) with the participation of SGLT-1 and Na^+^/K^+^ ATPase. An increase in [ATP]_i_ levels not only closed the K_ATP_ openers-induced channels openings but also provided the necessary phosphate groups for protein phosphorylation. Phosphorylation (inactivation) of PP2A-Tyr^307^ probably occurs at a site downstream of AMPKα-Thr^172^ phosphorylation (Figure 10).
